# Radionuclides’ Recovery from Seawater Using FIC and FIC A Sorbents

**DOI:** 10.3390/ma16114181

**Published:** 2023-06-04

**Authors:** Nikolay A. Bezhin, Vitaliy V. Milyutin, Natalia V. Kuzmenkova, Iuliia G. Shibetskaia, Ol’ga N. Kozlovskaia, Evgeniy V. Slizchenko, Victoria A. Razina, Ivan G. Tananaev

**Affiliations:** 1 Department of Biogeochemistry, Marine Hydrophysical Institute, Russian Academy of Sciences (MHI RAS), Kapitanskaya Str., 2, 299011 Sevastopol, Russia; nickbezhin@yandex.ru (N.A.B.); iuliia.shibetskaia@gmail.com (I.G.S.); o.n.kozlovska@gmail.com (O.N.K.); evgeniy774@gmail.com (E.V.S.); razina.v@mail.ru (V.A.R.); 2 Department of Chemistry and Chemical Engineering, Sevastopol State University, Universitetskaya Str., 33, 299053 Sevastopol, Russia; 3 Laboratory of Chromatography of Radioactive Elements, Frumkin Institute of Physical Chemistry and Electrochemistry, Russian Academy of Sciences (IPCE RAS), 31 Leninsky Prospect, 4, 119071 Moscow, Russia; vmilyutin@mail.ru; 4 Department of Radiochemistry, Lomonosov Moscow State University, 1 Leninskiye Gory, 3, 119991 Moscow, Russia; kuzmenkova213@gmail.com; 5 Radiochemistry Laboratory, Vernadsky Institute of Geochemistry and Analytical Chemistry, Russian Academy of Sciences (GEOKHI RAS), Kosygin Str., 19, 119991 Moscow, Russia

**Keywords:** FIC, radionuclides, seawater, sorption, dynamics, kinetics, isotherm

## Abstract

To solve radioecological and oceanological problems (estimate the vertical transport, flows of particulate organic carbon, phosphorus biodynamics, submarine groundwater discharge, etc.), it is necessary to determine the natural values of the radionuclides’ activity in seawater and particulate matter. For the first time, the radionuclides’ sorption from seawater was studied using sorbents based on activated carbon modified with iron(III) ferrocyanide (FIC) and based on activated carbon modified with iron(III) hydroxide (FIC A—activated FIC) obtained by FIC sorbent treatment with sodium hydroxide solution. The possibility of trace amounts of phosphorus, beryllium, and cesium recovery in laboratory conditions has been investigated. Distribution coefficients, dynamic, and total dynamic exchange capacities were determined. The physicochemical regularities (isotherm and kinetics) of sorption have been studied. The results obtained are characterized via Langmuir, Freindlich, and Dubinin–Radushkevich isotherm equations, as well as pseudo-first and pseudo-second-order kinetic models, intraparticle diffusion, and the Elovich model. Under expeditionary conditions, the sorption efficiency of ^137^Cs using FIC sorbent, ^7^Be, ^32^P, and ^33^P—using FIC A sorbent with a single-column method by adding a stable tracer, as well as the sorption efficiency of radionuclides ^210^Pb and ^234^Th with their natural content by FIC A sorbent in a two-column mode from large volumes of seawater was assessed. High values of efficiency of their recovery by the studied sorbents were achieved.

## 1. Introduction

The search for new materials for assessing and monitoring the pollution of natural waters with various pollutants [1], bacteria [2], and radionuclides (^60^Co [3], ^90^Sr [4], ^137^Cs [5], etc.) is an urgent task. Sorption processes have been used to recover radionuclides from seawater for decades. Selective sorbents make it possible to extract and concentrate radionuclides from large volumes of natural water rapidly, which gives the opportunity to largely simplify the analytical procedure compared to co-deposition processes.

Determination of the natural values of the radionuclides activity in seawater and particulate matter is used for radioecological monitoring (^137^Cs [6], ^90^Sr [7]) and for the study of various oceanological processes. They include vertical transport (^7^Be [8], ^32^P [9]), determination of sedimentation parameters and flows of particulate organic carbon (^210^Pb and ^210^Po [10], ^234^Th [11]), phosphorus biodynamics (^32^P, ^33^P) [12], submarine groundwater discharge (^223^Ra, ^224^Ra, ^226^Ra, ^228^Ra) [13], etc.

Sorbents based on ammonium phosphomolybdate [14] and transition metal hexacyanoferrates(II) [15] are most widely used for the recovery of ^137^Cs from seawater. Ammonium phosphomolybdate is impregnated onto various supports: polyacrylonitrile fiber (AMP-PAN) [14], SiO_2_ (AMP-SiO_2_) [16], and others. Transition metal hexacyanoferrates(II) can be used to sorb ^137^Cs from seawater in the solid form [17] or deposited on support: polyacrylonitrile fiber (KNiFC-PAN) [14], SiO_2_ (FSS [15], KCFC-SiO_2_ [16]), cellulose (Anfezh) [18], chitosan (CFC Zn-K, CFC Cu-K, CFC Ni-K) [19], hydrated zirconium dioxide (Thermoxide 35) [20], and others. The studies of sorbents based on transition metal hexacyanoferrates(II) indicate that the parameters of cesium sorption largely depend on the type of transition metal in the composition of the inorganic phase. Thus, in [21,22], the series of sorbents capacity decrease containing various transition metals concerning cesium are given. However, the decrease series obtained differ; the authors explain this discrepancy by the peculiarities of the formation of the sorption-active phase, the properties of which depend on the method of sorbent obtaining [22]. Therefore, it is necessary to carry out a series of comparative experiments for a real assessment of the effectiveness of these sorbents.

For direct preconcentration of ^210^Pb and ^210^Po from seawater, using sorbents based on manganese compounds is suggested: magnetite impregnated with 14% MnO_2_ [23], chemisorbents based on MnO_2_ [24], cartridges impregnated with manganese oxyhydroxide [25]. The latter showed a high sorption efficiency (96.5 ± 2.5%) of ^210^Pb and ^210^Po from 950 to 2000 L of seawater, but [25] does not give the conditions of the sorption process (seawater transmission rate and mass or volume of the sorbent). In [26], it was proposed to extract ^210^Pb with a fiber impregnated with Fe(OH)_3_. However, to achieve a high sorption efficiency, the authors propose passing seawater through the sorbent at a rate of only 50–60 mL/min, which is unacceptable under expeditionary conditions due to the long amount of time it takes to treat a single sample and, as a result, the need to have a large number of containers to obtain the data on the vertical distribution of these radionuclides concentration with high spatial resolution. In our work [27], we achieved a high sorption efficiency of ^210^Pb from seawater at a flow rate of 1 L/min using our sorbent based on Fe(OH)_3_. To separate ^210^Pb and ^210^Po from the accompanying radionuclides, extraction chromatographic sorbents based on crown ethers Sr Resin [28] and Pb Resin [29] were also proposed. Effective analogs of these sorbents based on an organofluoride diluent were obtained [30].

For the recovery of radium and thorium isotopes from seawater, sorbents based on mixed oxides of manganese are most often used. Membrane filters [31], polypropylene cartridges [25], acrylate fiber [32], and cellulose fiber [32] are used as MnO_2_ supports, as well as granular MnO_2_ without any support [24,33]. The sorbent based on acrylic fiber and MnO_2_ has received the widest application for the preconcentration of radium and thorium isotopes. In addition, the possibility of ^234^Th recovery from seawater with sorbents based on Fe(OH)_3_ was reported in [9]. It was successfully used by us [27], and a high sorption efficiency of ^234^Th was defined.

For the recovery of cosmogenic isotopes, ^7^Be and ^32^P, ^33^P aluminum oxide (Silker method) [34] and iron(III) hydroxide impregnated into polypropylene cartridges [35] or fibers [36] are used. The disadvantages of aluminum oxide are low recovery rates (about 50–60%), and impregnated polypropylene cartridges have high hydrodynamic resistance. In addition, the sorption component is gradually washed out from polypropylene cartridges impregnated with iron(III) hydroxide, which misrepresents the results. It is optimal to use an impregnated fiber [27]. Additionally, note that several researchers suggest using sorbents based on manganese dioxide to sorb ^7^Be [24].

The authors of [37] provide more details on the use of various sorption materials for the radionuclides’ recovery from the seawater, along with quantitative sorption characteristics.

Thus, sorbents based on iron(III) hydroxide can recover ^7^Be, ^32^P, ^33^P, ^210^Pb, ^210^Po, and ^234^Th isotopes from seawater and sorbents based on manganese dioxide are effective in recovering ^7^Be, ^210^Pb, ^210^Po, ^232^Ra, ^233^Ra, ^234^Ra, ^236^Ra, ^234^Th. However, only radionuclides that are determined by gamma spectrometry without radiochemical preparation can be simultaneously analyzed in sorbents after extraction: ^7^Be [38], ^137^Cs [39], ^210^Pb [40], ^226^Ra and ^228^Ra [41], ^234^Th [42]. The RaDeCC (Radium Delayed Coincidence Counter) systems are used to determine short-lived ^223^Ra and ^224^Ra isotopes [27]. To determine ^32^P, ^33^P, and ^210^Po, it is necessary to carry out independent radiochemical procedures, i.e., the samples need to be duplicated. After that, the activity of ^32^P and ^33^P is measured using β-spectrometry [31] and ^210^Po—α-spectrometry [29].

At present, most of the sorbents mentioned above are used in marine radiochemistry, but the search for the most sorption-efficient materials that provide extensive radionuclides recovery for solving oceanological and radioecological tasks is ongoing.

Due to its developed porous structure, activated carbon has become widely used as the carrier for the production of sorbents used in marine radiochemistry. By impregnating it with various modifiers, several sorbents were obtained for the recovery of different radionuclides from seawater. Thus, to extract uranium from seawater, activated carbon is used without modifiers [43] and with modification by polyethyleneimine [44]. When activated carbon is modified with KMnO_4_ solution, the MnO_2_·*x*H_2_O phase forms on its surface, which effectively absorbs transition metal radionuclides from seawater, including ^54^Mn and ^60^Co [45].

In this paper, for the first time, we present the results of a comprehensive study of sorbents based on activated carbon modified with iron(III) ferrocyanide (FIC) and based on activated carbon modified with iron(III) hydroxide (FIC A—activated FIC), obtained by FIC sorbent treatment with sodium hydroxide solution.

## 2. Materials and Methods

### 2.1. Materials

Cesium nitrate (analytically pure grade), potassium dihydrogen phosphate (analytically pure grade), and beryllium sulfate (analytically pure grade) produced by AO ReaKhim LLC (Moscow, Russia) were used as additives. Aluminon (analytically pure grade), ammonium acetate (analytically pure grade), ethylenediaminetetraacetic acid disodium salt (analytically pure grade), ammonium molybdate (analytically pure grade), sulfuric acid (analytically pure grade), potassium antimonate (analytically pure grade), ascorbic acid (analytically pure grade) produced by AO ReaKhim LLC (Moscow, Russia), and gum Arabic produced by Alland & Robert S.A. (Port-Mort, France) were used to determine the concentration of stable phosphorus and beryllium. To prepare the calibration solutions, standard samples of solutions were used: cesium—ISS (interstate standard sample) 0160:2004, beryllium—ISS 0352:2002 (LLC Ormet, Yekaterinburg, Russia).

The generalized composition of the used Black Sea water is given in [46].

Table 1 presents the main characteristics of FIC and FIC A sorbents. To obtain FIC A sorbent, FIC sorbent was activated to convert iron(III) ferrocyanide into iron(III) hydroxide. For this purpose, immediately before use, FIC was treated right in the column with a 0.5 mol/L sodium hydroxide solution (analytical grade, produced by AO ReaKhim LLC (Moscow, Russia) by passing 10 C.V. (column volumes) of the solution at a rate of 1 C.V./h. At the same time, iron(III) ferrocyanide transformed into active iron(III) hydroxide by reaction (Equation (1)) and became a good collector for ^7^Be, ^32^P, ^33^P, ^210^Pb, ^210^Po, ^234^Th radionuclides:

Fe_4_[Fe(CN)_6_]_3_ + 12NaOH → 4Fe(OH)_3_ + 3Na_4_[Fe(CN)_6_] (1)

### 2.2. IR Spectroscopy of Sorbents

To assess the transition of iron(III) ferrocyanide in FIC sorbent upon activation into iron(III) hydroxide in FIC A sorbent, the IR spectra of the sorbents were recorded using an InfraLUM FT-08 infrared Fourier spectrometer (Lyumex-Marketing LLC, St. Petersburg, Russia) using KBr pellets and processed with the SpectraLUM software v. 2.0.1.278 package with the connected thematic spectra libraries in JCamp formats developed by S.T. Japan-Europe specifically for the software of this device.

**Figure 1:**
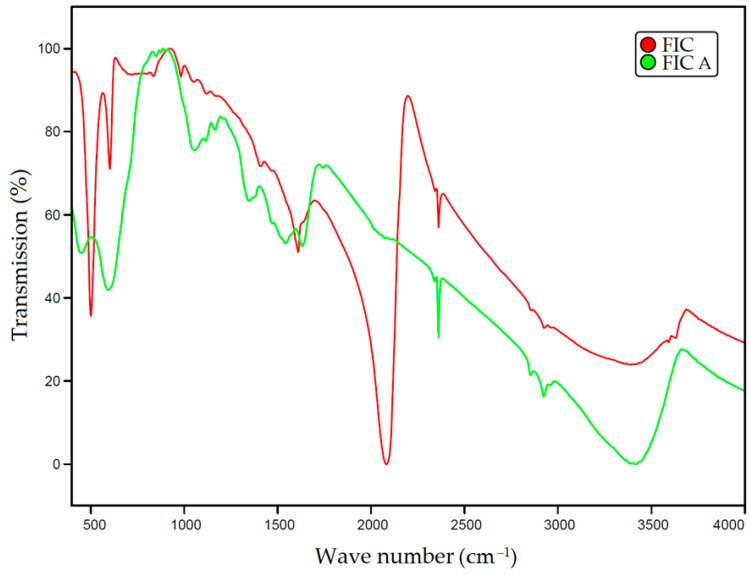
IR spectra of sorbents FIC and FIC A.

Figure 1 shows the obtained IR spectra of the FIC and FIC A sorbents. The IR spectrum of FIC sorbent clearly shows the peaks in the range 2000–2150 cm^−1^ corresponding to the C≡N group coordinated with the metal, which indicates the presence of the complex ion [Fe(CN)_6_]^4−^ [47]. After the activation of the sorbent with alkali, these peaks disappear. The activated sorbent contains peaks at 448, 1055, and 1082 cm^–1^, which can be attributed to vibrations of the Fe-OH bond [48].

### 2.3. Sorption Laboratory Research

In studies under laboratory conditions, cesium nitrate was added to seawater during the extraction of cesium, the additive for phosphorus was potassium dihydrogen phosphate, for beryllium—beryllium sulfate until reaching the concentrations for cesium, phosphorus, and beryllium—20; 0.1 and 0.3 mg/L, respectively [27,46]. The temperature of the sorption experiments was 20 °C.

Determination of distribution coefficients of cesium, phosphorus, and beryllium was carried out according to the unified procedure proposed in [49] by mixing 0.1 g of the sorbent with 20 mL of prepared seawater for 48 h. After that, the resulting mixtures were separated by filtration.

In dynamic experiments, prepared seawater was passed through a column with an inner diameter of 1 cm, filled with 3 g of the sorbent at various rates (3, 6, 15, 30 mL/min) in a manner similar to [46] using a LongerPump WT600-2J peristaltic pump (Longer Precision Pump Co., Baoding, China). After the column, the filtrates were collected by fractions and analyzed.

Sorption kinetics of cesium, phosphorus, and beryllium were determined in a similar manner to that described in [50] by mixing 0.1 g of the sorbent with 10 mL of prepared seawater for various periods. Then, the mixture was separated by filtration.

Sorption isotherms of cesium, phosphorus, and beryllium were studied in a similar manner to that described in [50] by mixing 0.1 g of the sorbent with 10 mL of prepared sea water with the addition of various concentrations of stable cesium, phosphorus, and beryllium for 48 h. After that, the mixture was separated by filtration.

### 2.4. Determination of the Cesium, Phosphorus, and Beryllium Concentration in Solution and Quantitative Parameters of Sorption

The concentration of cesium in solutions was determined using a KVANT-2 atomic absorption spectrophotometer (LLC Kortek, Moscow, Russia) in an air-acetylene flame in the emission mode at a wavelength of 852.1 nm. Calibration solutions with cesium concentrations of 5, 10, and 20 mg/L were prepared using seawater and ISS Cs 0160:2004. The error in determining cesium on an atomic absorption spectrophotometer averaged 1% and did not exceed 2%.

The concentration of beryllium in solutions was determined using a KFK-3-01 photometer (JSC Zagorsk Optical and Mechanical Plant, Sergiev Posad, Russia) according to the method described in [51]. During the procedure, an aluminon solution was prepared by dissolving 0.5 g of aluminon, 140 g of ammonium acetate, and 10 g of gum Arabic in distilled water, after which the volume of the solution was brought up to 1 L with distilled water. The resulting solution was filtered. A total of 5 mL of the disodium salt of ethylenediaminetetraacetic acid solution (5 g in 100 mL of water) and 10 mL of aluminon solution were added to 25 mL of the analyzed solution in a 50 mL volumetric flask. The mixture was heated for 10 min in a water bath, quickly cooled to room temperature (under cold water flow), and diluted with distilled water to the mark. Calibration solutions with beryllium concentrations of 0.1, 0.2, and 0.4 mg/L were prepared using ISS Be 0352:2002 and with the same reagents as the analyzed samples. Optical density was measured at 536 nm in two-centimeter cuvette using a blank sample prepared with the same reagents as for the analyzed samples. The relative error in the determination of beryllium was 2–4%.

The concentration of phosphorus in solutions was determined using a KFK-3-01 photometer (JSC Zagorsk Optical and Mechanical Plant, Sergiev Posad, Russia) according to the method described in [52]. The mixed reagent was prepared as follows: 50 mL of 2.5 mol/L sulfuric acid, 10 mL of potassium antimonate solution, and 20 mL of ammonium molybdate solution were mixed, and then 20 mL of ascorbic acid was added. A total of 10 mL of the analyzed samples was transferred into 15 mL plastic tubes, 1 mL of the mixed reagent was added to each, and the tubes were closed with stoppers and mixed. Calibration solutions with phosphorus concentrations of 1, 4, and 8 µmol/L were prepared using a standard solution of potassium dihydrophosphate and mixed reagents, similar to the analyzed samples. After 10 min, the optical density of the colored solutions was measured at a wavelength of 880 nm in a five-centimeter cuvette using a blank sample prepared with reagents, as well as the analyzed samples. The relative error in the phosphorus determination was 1.5–2%.

The distribution coefficient (*K_d_*, mL/g), dynamic exchange capacity (DEC, mg/g), and the total dynamic exchange capacity (TDEC, mg/g) were determined according to the equations given in [46]. The degree of recovery (*R*, %) and the sorbents capacity (*q*, mg/g) were determined according to the equations given in [30].

### 2.5. Evaluation of the Sorption Efficiency

To study the radionuclides’ sorption using the FIC and FIC A sorbents, seawater samples were taken during a 121-day (19 April–14 May 2022) “R/V Professor Vodyanitsky” cruise (Centre of collective usage R/V “Professor Vodyanitsky” A.O. Kovalevsky Institute of Biology of the Southern Seas of RAS) along the southern coast of Crimea.

The sorption of ^137^Cs was achieved by a single-column method by passing 250 L of seawater at different rates using a LongerPump WT600-2J peristaltic pump (Longer Precision Pump Co., Baoding, China) through a column filled with 50 mL of FIC sorbent. Stable cesium was added to the seawater sample as an output tracer to a concentration of 2.5 mg/L.

The sorption of ^7^Be, ^32^P, and ^33^P was carried out using a single-column method by passing 250 L of seawater at different rates using a Longer Pump WT600-2J peristaltic pump (Longer Precision Pump Co., Baoding, China) through a column filled with 50 mL of FIC A sorbent. Potassium dihydrogen phosphate and beryllium sulfate were added to the seawater sample as output tracers in the concentrations of 0.1 and 0.3 mg/L, respectively.

To estimate the yield, every 10–50 L, the seawater passed through the sorbent was taken. The sorption efficiency of ^7^Be, ^32^P, ^33^P, and ^137^Cs from seawater was determined by the equations given in refs. [36,53].

The sorption of ^210^Pb and ^234^Th was carried out using a two-column method by passing 250 L of seawater through a system of two columns, each filled with 50 mL of FIC A sorbent, at different rates. After passing the seawater, the sorbent was dried and placed in Petri dishes. The activity of radionuclides was measured using a CANBERRA low-background semiconductor γ-spectrometer with a high-purity germanium detector GC3020 (Canberra Industries, Meriden, CT, USA) for at least 48 h. In this case, the relative measurement error was 16–19% for ^210^Pb and 13–14% for ^234^Th. The sorption efficiency of ^210^Pb and ^234^Th from seawater was determined by the equations given in [54].

## 3. Results and Discussion

### 3.1. Distribution Coefficients of Cesium, Phosphorus, and Beryllium

During laboratory tests, the parameters for the extraction of cesium by FIC sorbent and phosphorus and beryllium by FIC A sorbent from the seawater were determined under static and dynamic conditions.

The results of the evaluation of distribution coefficients are shown in Table 2.

It is clear that FIC sorbent effectively extracts cesium from seawater. The determined values of the distribution coefficient correlate with the distribution coefficients of sorbents based on nickel-potassium ferrocyanide Niket (*K_d_* = (1.6 ± 0.2)∙10^4^ mL/g) and FSS (*K_d_* = (1.1 ± 0.3)∙10^4^ mL/g) obtained in our previous comprehensive study of several sorbents for cesium sorption from seawater [46].

FIC A shows high parameters for phosphorus recovery from seawater comparable to the results defined for the previously obtained Fe-SF (Fe-Sodium Ferrate) sorbent based on Fe(OH)_3_ (*K_d_* = (4.1 ± 0.3)∙10^3^ mL/g), which was obtained using the prepared sodium ferrate [27]. The distribution coefficient of beryllium is lower than that defined for the Fe-SF sorbent but comparable with the values for our Fe-NH (Fe-Non-Hydrolyzed) sorbent (*K_d_* = 520 ± 70 mL/g) obtained using non-hydrolyzed PAN and precipitation of Fe(OH)_3_ with ammonia.

### 3.2. Sorption Dynamics

**Figure 2:**
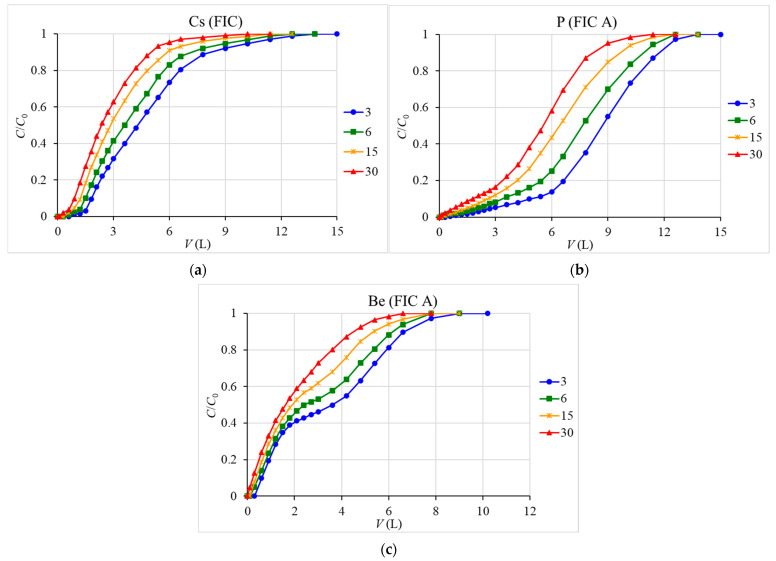
Output sorption curves of cesium (**a**), phosphorus (**b**), and beryllium (**c**) at different seawater flow rates (3, 6, 15, and 30 mL/min), where *C*_0_ is the feed extractable element concentration in the solution, mg/L; *C* is the residual extractable element concentration in the emerging filtrate, mg/L, *V* is the solution volume, liters (L).

Figure 2 shows the output sorption curves of cesium, phosphorus, and beryllium under dynamic conditions at different seawater transmission rates. With an increase in the solution flow rate, the number of leaked ions increased due to a decrease in the contact time of the passed solution with the sorbent. The maximum volume of passed seawater with the addition of the studied stable isotope, depending on the transmission rate, was 12.6–15 L for FIC sorbent when recovering cesium, 12.6–15 L for FIC A sorbent when recovering phosphorus, and 7.8–10.2 L when recovering beryllium.

Based on the experimental data obtained, the values of DEC and TDEC of the studied sorbents were calculated; they are presented in Table 3. In terms of DEC and TDEC (5.61 and 27.5 mg/g, respectively, at a flow rate of 3 mL/min), FIC sorbent proved to be as good as effective sorbents such as Uniket (5.62 and 77.7 mg/g) and Anfezh (1.87 and 27.3 mg/g) [46]. In terms of DEC and TDEC for phosphorus (0.027 and 0.358 mg/g), FIC A was only slightly inferior to the best fibrous sorbent Fe-H (Fe-Hydrolyzed) based on iron(III) hydroxide (0.0375 and 0.394 mg/g) [27] was obtained using pre-hydrolyzed PAN with precipitation of iron(III) hydroxide with ammonia. At the same time, for FIC A sorbent, the DEC and TDEC values for beryllium (0.0132 and 0.0716 mg/g) are an order lower than that for Fe-H (0.0676 and 0.51 mg/g), but higher than that for aluminum oxide (0.00346 and 0.0444 mg/g) [27].

### 3.3. Sorption Kinetics

**Figure 3:**
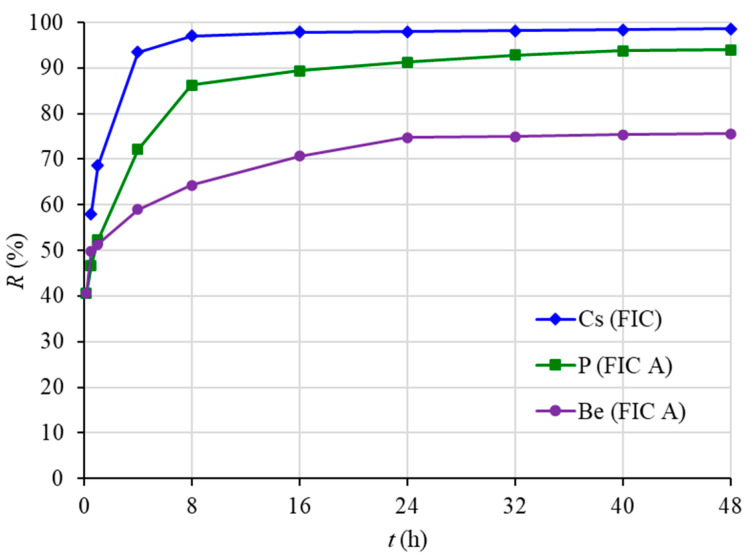
Dependences of the degree of recovery (*R*) on the sorption time (*t*).

Figure 3 shows the results of determining the dependence of the degree of recovery of cesium, phosphorus, and beryllium on the sorption time. It has been determined that more than 90% of cesium is recovered by FIC sorbent in 4 h, and the sorption equilibrium is established in 16 h. In general, this corresponds to the data regarding the sorption time for ferrocyanide sorbents. Thus, when extracting cesium with the Niket sorbent, equilibrium is reached in 16 h, while with Uniket and FSS sorbents, equilibrium is reached in 24 h [55].

More than 90% of phosphorus is recovered by FIC A in 24 h, and sorption equilibrium is established in 40 h. A total of 75% of beryllium is recovered by FIC A in 24 h, and the sorption equilibrium for beryllium is established in 40 h. This corresponds to the data on the sorption times for phosphorus and beryllium for similar sorbents based on iron(III) hydroxide [56].

The obtained experimental parameters for the sorption of the studied elements were described using kinetic models of the pseudo-first [50] and pseudo-second [57] orders, intraparticle diffusion [57], and the Elovich model [58].

The established theoretical values of the equilibrium capacity according to the pseudo-second order model correlate with the obtained experimental values of the equilibrium capacity (Table 4).

Generally, the pseudo-second order model, which takes into account not only sorbate–sorbent interactions but also intermolecular interactions of adsorbed substances, accurately describes the obtained experimental data. Thus, the chemical exchange reaction limits the sorption process [57].

### 3.4. Sorption Isotherm

**Figure 4:**
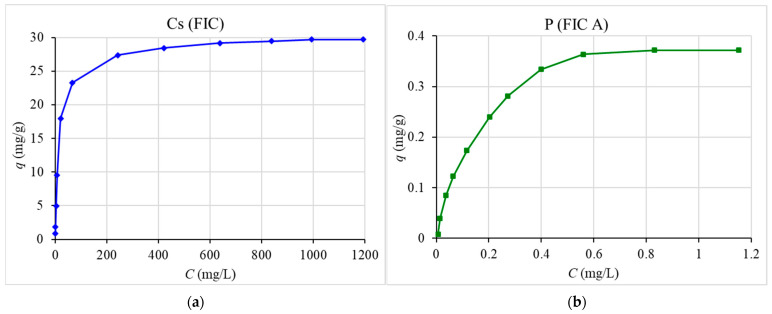

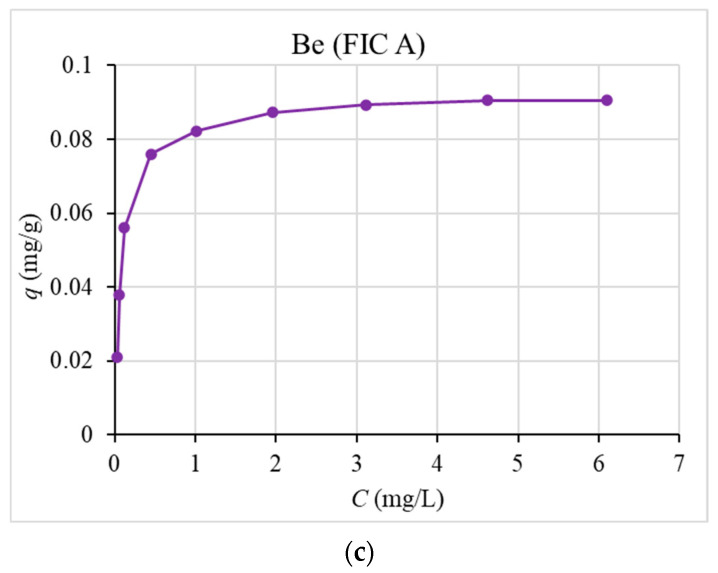
Sorption isotherms of cesium (**a**), phosphorus (**b**), and beryllium (**c**), where *q* is the capacity of the sorbent, mg/g; *C* is the equilibrium concentration, mg/L.

Figure 4 shows the results derived from studying the cesium, phosphorus, and beryllium sorption isotherms.

It can be seen that the maximum capacity of FIC sorbent for cesium is established at an equilibrium concentration of cesium in a solution of more than 900 mg/L. For FIC A for phosphorus, this occurs when the equilibrium concentration of phosphorus is more than 0.6 mg/L, and for beryllium, the equilibrium concentration of beryllium needs to be more than 4 mg/L. This corresponds to the data obtained for analogous sorbents based on transition metal ferrocyanides by the cesium sorption [55] and based on iron(III) hydroxide by the phosphorus and beryllium sorption [56].

The defined maximum capacity of FIC sorbent for cesium (29.7 mg/g) is somewhat higher than that of the Anfezh sorbent (27.0 mg/g) [46], which has a good reputation in the USA [59] and Japan [60] for the sorption of ^137^Cs from radioactive waste based on seawater. The maximum capacity of FIC A for phosphorus (0.372 mg/g) is comparable to the capacity for Fe-H sorbent (0.425 mg/g) [34].

The obtained experimental parameters for the sorption of the studied elements were processed using the Langmuir [50], Freindlich [50], and Dubinin–Radushkevich [61] sorption isotherm equations. The obtained parameters are presented in Table 5.

The calculated values of the maximum capacity of the studied sorbents for cesium, phosphorus, and beryllium obtained from the linearized Langmuir isotherm equation are in good agreement with the experimental capacity values, which indicates that the description of the sorption of these elements by the Langmuir sorption isotherm equation is adequate.

The Langmuir model describes adsorption on a monomolecular layer well, which corresponds to the morphology of the studied sorption materials obtained by modifying the support surface.

### 3.5. Sorption Efficiency

**Figure 5:**
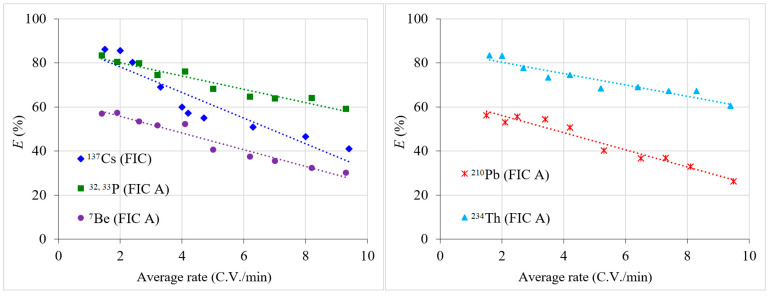
Dependence of the sorption efficiency (*E*) of radionuclides on the average rate of seawater transmission.

Figure 5 shows the results of expeditionary experiments on the sorption of radionuclides from large-volume samples.

To determine the sorption efficiency of ^7^Be, ^32^P, ^33^P, and ^137^Cs, the single-column method was used with the addition of a stable tracer to seawater samples. The study of the sorption efficiency of ^210^Pb and ^234^Th was carried out on their natural content using the two-column method.

It is clear that FIC sorbent effectively extracts ^137^Cs from large volumes of seawater at transmission rates of 1.5–4 C.V./min (*E* = 60–86.1%). Thus, in expeditionary research, the FIC sorbent is more efficient than the Anfezh sorbent and extracts ^137^Cs in a way that is on par with the Uniket sorbent [53].

FIC A sorbent can be successfully used to sorb ^32^P, ^33^P, and ^234^Th at a transmission rate of 1.5–8 C.V./min, as well as ^7^Be and ^210^Pb at a transmission rate of 1.5–4 C.V./min. FIC A shows a high sorption efficiency of ^32^P and ^33^P (*E* = 59.1–83.4%) from large volumes of seawater—higher than the Fe-SF sorbent (*E* = 45.4–74%) [12]. Although the sorption efficiency for ^7^Be and ^210^Pb is within 50–60% at a transmission rate of 1.5–4 C.V./min, this, on the contrary, makes it possible to determine the activity of these radionuclides on the second adsorber more accurately.

The sorption efficiency of the studied radionuclides by FIC and FIC A sorbents is determined by their active component: iron(III) ferrocyanide and iron(III) hydroxide, respectively. So, transition metal ferrocyanides are effective collectors of cesium and iron(III) hydroxide are effective collectors of phosphorus, beryllium, lead, and thorium.

The sorption mechanisms are as follows:

- Cesium on FIC sorbent by reaction Equation (2):

12Cs^+^ + Fe_4_[Fe(CN)_6_]_3_ = 4Fe^3+^ + 3Cs_4_[Fe(CN)_6_] (2)

- Beryllium or lead on FIC A sorbent by reaction Equation (3):

3Me^2+^ + 2Fe(OH)_3_ = 2Fe^3+^ + 3Me(OH)_2_, (3)

- Thorium on FIC A sorbent by reaction Equation (4):

3Th^4+^ + 4Fe(OH)_3_ = 4Fe^3+^ + 3Th(OH)_4_, (4)

- Phosphorus or lead on FIC A sorbent by reaction Equation (5):

Fe(OH)_3_ + PO_4_^3−^ = FePO_4_ + 3OH^−^. (5)

It is also possible to carry out the complex sorption of radionuclides by sequentially passing water through columns filled with FIC and FIC A sorbents since these sorbents recover different radionuclides (FIC sorbs ^137^Cs, FIK A—^7^Be, ^32^P, ^33^P, ^210^Pb, ^234^Th).

These sorbents can be successfully used to solve radioecological (assessment and search for sources of ^137^Cs input into marine ecosystems [62]) and oceanological problems (estimates of vertical transport [63], phosphorus biodynamics [64], suspended organic carbon fluxes [65]) through the use of radiotracers methods.

## 4. Conclusions

For the first time, FIC and FIC A sorbents were proposed for the sorption of radionuclides of various origins from seawater. These sorbents consist of activated carbon modified with iron(III) ferrocyanide and iron(III) hydroxide, respectively.

Comprehensive laboratory and expeditionary tests of these sorbents for the recovery of cesium, phosphorus, and beryllium from seawater were carried out to determine the best recovery conditions when using these sorbents.

The results show high distribution coefficients for cesium ((1.3 ± 0.2)∙10^4^ mL/g) and phosphorus ((3.6 ± 0.2)∙10^3^ mL/g)—comparable to the most effective sorption materials for seawater. Isotherms, output, and kinetic curves of sorption were plotted. The values of dynamic and total dynamic capacity for the studied sorbents, the dependence of the degree of recovery on the time of sorption, and the capacity of the sorbent on the equilibrium concentration of the recovered element in solution were obtained. The comparability of the obtained experimental values with the theoretical data was determined using the pseudo-second order model (*r*^2^ > 0.999), and the Langmuir sorption isotherm equation (*r*^2^ > 0.996) was determined.

The FIC sorbent proved that it could be successfully used to sorb ^137^Cs from seawater at a transmission rate of 1.5–4 C.V./min, and the FIC A sorbent proved useful for the sorption of ^32^P, ^33^P, and ^234^Th at a transmission rate of 1.5–8 C.V./min, as well as ^7^Be and ^210^Pb at a transmission rate of 1.5–4 C.V./min.

Thus, the studied sorbents can be used to sorb concentrate radionuclides from seawater. Due to their sequential arrangement, the studied sorbents can also be used for complex sorption to solve radioecological and oceanological problems, which we plan to do in the future.

## Figures and Tables

**Table 1:** The main characteristics of sorbents FIC and FIC A.

Sorbent	Manufacturer	Appearance	Particle Size, mm	Bulk Weight, g/cm^3^	Sorbent Composition	
					Support	Sorption-Active Phase: Content (Mass %)
FIC	Frumkin Institute of Physical Chemistry and Electrochemistry, Russian Academy of Sciences	dark blue irregular granules	0.1–1.0	0.25–0.4	activated carbon	iron(III) ferrocyanide; not less than 10
FIC A		black irregular granules				iron(III) hydroxide; not less than 10

**Table 2:** Distribution coefficients of cesium, phosphorus, and beryllium.

Sorbent	FIC	FIC A	
Recovered element	Cs	P	Be
*K_d_*, mL/g	(1.3 ± 0.2)∙10^4^	(3.6 ± 0.2)∙10^3^	510 ± 45

**Table 3:** DEC and TDEC values for cesium, phosphorus, and beryllium.

Sorbent	Recovered Element	Parameter	Flow Rate, mL/min			
			3	6	15	30
FIC	Cs	DEC, mg/g	5.61	3.73	2.80	1.87
		TDEC, mg/g	27.5	23.5	19.1	15.8
FIC A	P	DEC, mg/g	0.027	0.018	0.009	0.0045
		TDEC, mg/g	0.358	0.313	0.265	0.224
	Be	DEC, mg/g	0.0132	0.0088	0.0044	0.0022
		TDEC, mg/g	0.0716	0.0616	0.0509	0.0409

**Table 4:** Parameters of kinetic models.

Sorbent	Recovered Element	Intraparticle Diffusion *			Pseudo-First-Order *			Pseudo-Second-Order *			Elovich Model *			*q_e exp_*, mg/g
		*K_I_*, mg/(g∙h^0.5^)	*c*, mg/g	*r* ^2^	*K*_1_, h^−1^	*g_e_*, mg/g	*r* ^2^	*K*_2_, g/(mg∙h)	*g_e_*, mg/g	*r* ^2^	*α*, g/(mg∙h)	*β*, g/mg	*r* ^2^	
FIC	Cs	0.129	1.14	0.648	0.128	0.349	0.810	1.87	1.86	1.00	178	5.45	0.899	1.84
FIC A	P	0.0030	0.0124	0.805	0.130	0.0147	0.959	34.8	0.0298	0.999	0.218	256	0.952	0.0293
	Be	0.0008	0.0075	0.897	0.126	0.0047	0.985	109	0.0123	0.999	4.45	1000	0.987	0.0122

* *K_I_* is the rate constant of intraparticle diffusion, mg/(g∙h^0.5^); *c* is the constant characterizing the contribution of the boundary layer, mg/g; *q_e_* is the equilibrium capacity of the sorbent, mg/g; *K*_1_ is pseudo-first order rate constant, h^–1^; *K*_2_ is pseudo-second order rate constant, g/(mg∙h); *α* is the initial sorption rate constant, g/(mg h); *β* is the desorption constant, g/mg; *r*^2^ is approximation confidence factor.

**Table 5:** Parameters of sorption isotherms.

Sorbent	Recovered Element	Langmuir Isotherm *			Freindlich Isotherm *			Dubinin–Radushkevich Isotherm *				*q_m exp_*, MΓ/Γ
		*q_m_*, mg/g	*K_L_*, L/mg	*r* ^2^	*K_F_*, mg/g	*n*	*r* ^2^	*q_m_*, mg/g	*β,* mol^2^/kJ^2^	*E,* kJ/mol	*r* ^2^	
FIC	Cs	29.9	0.067	0.999	4.18	3.18	0.901	25.4	0.011	6.65	0.959	29.7
FIC A	P	0.384	8.32	0.996	0.468	1.94	0.949	0.976	0.0096	7.22	0.957	0.372
	Be	0.091	13.1	0.999	0.075	5.95	0.866	0.101	0.0068	8.57	0.940	0.091

* *q_m_* is the maximum capacity of the sorbent, mg/g; *K_L_* is the Langmuir adsorption equilibrium constant, L/mg; *K_F_* is the Freundlich constant, mg/g; *n* is the empirical indicator of the heterogeneity of exchange centers; *β* is the constant associated with sorption energy, mol^2^/kJ^2^; *E* is the average free energy of sorption, kJ/mol; *r*^2^ is the approximation confidence factor.

## Data Availability

Not applicable.

## Abbreviations

AMP	ammonium phosphomolybdate
DEC	dynamic exchange capacity
Fe-H	Fe-Hydrolyzed (sorbent based on Fe(OH)_3_ was obtained using pre-hydrolyzed PAN with precipitation of Fe(OH)_3_ with ammonia)
Fe-NH	Fe-Non-Hydrolyzed (sorbent based on Fe(OH)_3_ was obtained using non-hydrolyzed PAN and precipitation of Fe(OH)_3_ with ammonia)
Fe-SF	Fe-Sodium Ferrate (sorbent based on Fe(OH)_3_ was obtained using the prepared sodium ferrate)
FIC	activated carbon modified with iron(III) ferrocyanide
FIC A	activated FIC (activated carbon modified with iron(III) hydroxide, obtained by FIC sorbent treatment with sodium hydroxide solution)
FSS	ferrocyanide-silicate sorbent
PAN	polyacrylonitrile
RaDeCC	Radium Delayed Coincidence Counter
TDEC	total dynamic exchange capacity
